# Economic burden of becoming a dentist in Thailand

**DOI:** 10.1038/s41405-023-00131-1

**Published:** 2023-02-10

**Authors:** Teerawat Tussanapirom, Prachya Siribal, Phiranat Trirattanaphinthusorn, Witchapat Kengtong, Piyada Gaewkhiew

**Affiliations:** 1 Department of Community Dentistry, Faculty of Dentistry, Mahidol University, Bangkok, Thailand; 2 Chalermprakiat Hospital, Buriram, Thailand; 3 Private Dental Clinic, Bangkok, Thailand; 4 Department of Oral Medicine, Faculty of Dentistry, Chulalongkorn University, Bangkok, Thailand

**Keywords:** Dental foundation training, Dental clinical teaching

## Abstract

**Objectives:**

To determine the overall estimated financial impact and related expenses incurred over the duration of the undergraduate Dental Degree in Thailand.

**Methods:**

A cross-sectional survey was conducted among all 658 dental undergraduates in Mahidol University, Thailand. Data was collected through a self-administered questionnaire, including the following information: (1) “Background and Demographics”: household income, hometown, residence during study and source(s) of any financial aid received; (2) “Living Expenses”: Living costs including food, transportation, rent, utility bills, and recreational expenses; (3) ”Education related expenses”: Including textbooks, stationeries, uniforms, and student activities fees. A cost-median was used as a baseline representation for the actual cost of each item. The mean differences of all expenses between groups before estimation was assessed by using the analysis of variance (ANOVA) method. The statistically significant differences were identified at *p* < 0.001.

**Results:**

The estimated adjusted cost of becoming a dentist in Thailand is THB1,265,027 (36,143.63 USD) for students living at home and THB1,823,027 (52,086.49 USD) for those renting accommodation. Students who rented accommodation incurred significantly higher yearly living expenses than those who were living at home. (*p* < 0.001). The majority of participants (78.4%) were in households having a middle-to-high socioeconomic status. Ninety-five percentages of the participants’ received 100% financial support from their families with no additional source of income, which reflects no real diversity in the socioeconomic background of Dental Degree students.

**Conclusion:**

The cost of a higher education Dental Degree in Thailand can be a significant barrier to entry and financial burden, especially for students from disadvantaged socioeconomic backgrounds. Government and Educational Policy makers need to pay more attention to this issue in order to provide equal opportunities for obtaining a University Dental Degree for all Thai students wishing to pursue this career path.

## Introduction

The financial burden for higher education is one of the main barriers in pursuing a Dentistry career path, especially for those coming from a socioeconomically disadvantaged background [1–3]. In Thailand, the government currently spends enormous amounts of money to subsidise public Universities with Dental Degree programs, resulting in tuition fees being very low compared to those at Private Universities [4]. The Government also issued a policy allowing students to obtain low-interest rate loans to pay for tuition fees [5]. However, the loan amounts are often not enough to fully cover the cost of tuition and living expenses, which for many average income Thai families, makes the total cost of education unaffordable.

Consequently, the high cost of education and related living expenses for University Degree can deprive many highly qualified students of an opportunity to pursue a Dental career coming from low-income families. These issues have been well-documented in other countries, including the U.S.A., U.K., Canada, and New Zealand [1, 6–8]. While the high costs of a Dental education has been long recognised as a burdening socioeconomic issue, nevertheless, until now, there has been no systematic study that aims to evaluate the financial burdens of dental students that were forced to choose other career paths due to the high cost of a University Dental Degree in Thailand.

In Thailand, over the past decade, eighteen dental schools have produced around 500–700 registered Dentists per year. The Faculty of Dentistry, Mahidol University, is one of Thailand’s most prominent dental schools and every year around 20% of all Dental School graduates are from Mahidol University.

The primary focus and goals of this study are to explore the magnitude of a Dental students’ financial burden and to generate greater public interest in these socioeconomic issues. Additionally, the aim of this study was to determine the financial profile of Mahidol’s Dental students and the financial expenses they incurred over the six-year duration of their undergraduate Dental Degree.

## Methods

In January of 2016, a cross-sectional survey was conducted among all 658 Dental undergraduate students. The expected minimum sample size was approximately 60% [9] of total population or 395 participants. All registered Mahidol Dental students (year 1–6) were invited to participate in this study. Students in other faculties, universities, alumni or university staff were not allowed to participate in this study. The eligible participants were contacted by a research team who were not lecturers. The self-administered paper-based questionnaire was distributed to those students after classes. All information provided was kept strictly confidential. Verbal and written consent was granted by the participants prior to filling out the questionnaire. All students had the right to terminate their participation at any time during this study.

We developed a questionnaire-based survey to gather information that would accurately document the dental students’ financial situation. The questionnaire was organised into two major sections: Background and Expenses.

The “Background” section required respondents to Indicate: The range of their household income, hometown (Urban, Rural) and residence during the study (Home, Rental accommodation), and any source(s) of financial support (Fully family support, Family support with a loan, Family support with scholarship).

The “Expenses” section required respondents to indicate the amounts they spent on: Living costs: including food, transportation, rent, other living costs (utility bills, laundry), along with any social and recreational expenses. Education-related expenses: including textbooks, stationary, extra laboratory equipment [3], uniforms, and student activities fees.

The expenses section was developed using expense categories from the ‘Thailand Household Socio-Economic Survey’ as a reference [10], and adjusted by a focus group discussion between twelve Mahidol dental students.

The Education Management Department, Faculty of Dentistry, Mahidol University, provided the data on tuition and license examination fees. The expenses are presented in Thai Baht (THB) and US dollar (USD) (THB35 = 1USD), which were adjusted by using the Purchasing Power Parity (PPP) provided by Big Mac Index 2016 [11].

A pilot study was conducted among 20 Dental students (using the questionnaire) who were not a part of this study, in order to test the acceptability and validity of the information. Following the feedback from the pilot study, there were no significant amendments made to the questionnaire. A final version of the questionnaire was distributed to all eligible participants.

### Ethical consideration

This study received ethical approval from the Faculty of Dentistry/Faculty of Pharmacy, Mahidol University, Institutional Review, Board (MU-DT/PY-IRB), project number 2015/DT076(COA. No. MU-DT/PY-IRB 2016/004.0701).

### Data analysis

The actual expense of Dental students can be challenging to assess. The living costs can vary greatly from student to student depending on their socioeconomic statuses (SES) and lifestyles. In order to avoid upward distorted financial cost data by the spending habits of wealthier students, we adopted a cost-median as a representation of the actual cost of each item.

The analysis of variance (ANOVA) was utilized in this study to assess the mean differences of all expense variables between student groups before estimation. For example, the expense of food between classes of students in the same type of accommodation was not significantly different, except for the first-year students (using different campuses). Therefore, estimations of food expenditure were calculated together with year 2–6 students living in the same accommodation type. In contrast, the first year’s expense for food was estimated separately. Linear regression was used to analyse the association between yearly expense and potential determinants.

The opportunity costs of an additional two years of study were also included in this study; this longer-than-normal duration of bachelor’s degree completion can be a significant burden for students’ families. All statistical analysis was conducted on SPSS version 18.0.

## Results

We received completed questionnaires from a total of 486 Dental undergraduates from the Faculty of Dentistry, Mahidol University (a 75.0% response rate). The majority of students were females (71.8%). 87.9% came from Bangkok Metropolitan Region and municipal areas. The majority of students were supported financially by their families, with a household income ranging between THB50,001 – 100,000 (1428–2857 USD) as represented in Table 1.

**Table 1:** Characteristics and Economic background (*N* = 486).

	*N*	%
Total	486	100%
Gender		
Male	137	28.2%
Female	349	71.8%
Place of residence		
Bangkok Metropolitan Region	310	63.8%
Provinces (Municipality)	117	24.1%
Suburb	59	12.1%
Student year		
Year 1	74	15.2%
Year 2	126	21.8%
Year 3	71	14.6%
Year 4	77	15.8%
Year 5	49	10.1%
Year 6	109	22.4%
Source of financing		
Fully Family	453	93.2%
Family support with Loan	12	2.5%
Family support with Scholarship	16	3.3%
N/A	5	1%
Household income (THB)		
<10,000 (>424 USD)	3	0.6%
10,000–15,000 (424–635 USD)	3	0.6%
15,001–30,000 (645.1–1,270 USD)	25	5.1%
30,001–50,000 (1,270.1–2,118 USD)	73	15.0%
50,001–100,000 (2,118.1–4,235 USD)	214	43.9%
>100,000 (>4235 USD)	168	34.5%

*THB* Thai Baht, *USD* US Dollar, *N/A* non- applicable.

### Financial support

All respondents received direct monetary support from their parents or relatives. Only twenty-three (23) out of the four-hundred-eighty-seven (487) respondents (5.0%) obtained partial financial aid from other sources. The most common source of additional financial support is from the Student Loan Fund (Low-interest rate student loans offered by the government). Other students received financial aid from partial scholarships, such as a scholarship from their parents’ workplaces or scholarships for a civil servant’s child (Table 2). The additional financial support received ranged from THB2,000 (57 USD) to THB60,000 (1714.29 USD) with a cost median of THB30,000 (857.14 USD) and an average amount of THB28,068 (1270 USD).

**Table 2:** Sources of Financial Support by Household Income (*N* = 23).

Household Income (THB)	Government Loan Fund	Others sources	Total
<10,000 (<424 USD)	1	0	1
10,000–15,000 (424–635 USD)	1	0	1
15,001–30,000 (645.1–1270 USD)	5	1	6
30,001–50,000 (1270.1–2118 USD)	1	5	6
50,001–100,000 (2118.1–4235 USD)	2	1	3
>100,000 (>4235 USD)	2	4	6
Total	12	11	23

*THB* Thai Baht, *USD* US Dollar.

### Yearly expense

We calculated the yearly expenses of a Dental Degree student by using the sum of the cost of living, tuition fees, other education-related expenses and license examination fees (for students years 4–6). The living costs and other education-related expenses were estimated by the sum of the median cost of all items in each category. Details of each item are described in Appendix 1. There are significant differences in daily spending between the students who live at home and the those who live in rental accommodation (Table 3). Therefore, we divided the Dental Degree students into two groups based on accommodation type during their years of study. Table 3 also shows the estimated yearly expenses of two dental student groups divided by accommodation type. Students who had to reside in a rental accommodation had significantly higher yearly expenses than those who lived at home (*p* < 0.001). Additionally, the association between yearly expense and type of accommodation was statistically significant both in crude and adjusted models with household income, hometown areas, year of education and gender (*p* < 0.001) (Table 4).

**Table 3:** Yearly expense of dental student (*N* = 486).

Year	Type of accommodation	Living cost		Tuition fee		Other education-related expense		License exam fee		Total yearly expense		Yearly expense: average Thais household
		THB	(USD)	THB	(USD)	THB	(USD)	THB	(USD)	THB	(USD)	(%)^†^
1	Home	N/A		N/A		N/A		N/A		N/A		N/A
	Rental	162,294	6873	29,550	1252	6638	281	0	0	198,482	8406	78.2%
	*P* value*									N/A		
2	Home	103,800	4396	28,000	1185	4000	169	0	0	135,800	5752	53.5%
	Rental	215,400	9123	28,000	1186	4000	169	0	0	247,400	10,479	97.5%
	*P* value*									<0.001		
3	Home	103,800	4396	27,600	1169	7195	305	0	0	138,595	5870	54.6%
	Rental	215,400	9123	27,600	1169	7195	305	0	0	250,195	10,597	98.6%
	*P* value*									<0.001		
4	Home	103,800	4396	29,000	1228	12,900	546	3000	127	148,700	6298	58.6%
	Rental	215,400	9123	29,000	1228	12,900	546	3000	127	260,300	11,024	102.6%
	*P* value*									<0.001		
5	Home	103,800	4396	28,600	1211	5150	218	0	0	137,550	5826	54.2%
	Rental	215,400	9123	28,600	1211	5150	218	0	0	249,150	10,552	98.2%
	*P* value*									<0.001		
6	Home	103,800	4396	29,800	1262	7300	309	5000	211	145,900	6179	57.5%
	Rental	215,400	9123	29,800	1262	7300	309	5000	211	257,500	10,906	101.5%
	*P* value*									<0.001		

*THB* Thai Baht, *USD* US Dollar, *N/A* non-applicable.

^†^Compared with average Thais household yearly expense form Thai National Household Survey 2016; conducted by National Statistic Office.

*t-test and ANOVA analysis for difference between home and rental accommodation groups by year of study and all participants, respectively.

**Table 4:** Models for the association between yearly expense and type of accommodation among dental students (*n* = 486).

	Model1		Model 2		Model 3		Model 4	
	Coeff	[95% CI]	Coeff	[95% CI]	Coeff	[95% CI]	Coeff	[95% CI]
Type of Accommodation								
Home	Ref	Ref	Ref	Ref	Ref	Ref	Ref	Ref
Rental	7299.19	[58,242.33, 87,756.04]**	77,243.35	[62,940.34, 91,546.3]**	73,765.9	[58,636.24, 8895.55]**	93,821.09	[78,799, 108,843.2]**

Model 2: Model 1 adjusted with household income; Model 3: Model 2 adjusted with hometown area; Model 4 adjusted household income, hometown area, year of education, and gender.

Linear regression; **p < 0.001.

^a^Linear regression.

Overall, the yearly expense of fourth and six-year students are relatively high compared to other classes due to an increase in other educational-related expenses (clinical uniforms for fourth-year and laboratory equipment costs for sixth-year students). Tuition fees can be considered a minor expense; they accounted for only 19.5–20.8% of the total yearly expenses for the student who lived at home and 11.41–14.89% of the total yearly expenses for those who lived in rental accommodation.

### Cost of becoming a dentist

This study estimated the total expense a Dental student has to pay for a six year degree is THB905,027 (25,857.91 USD) for students who mainly reside at home and THB1,463,027 (41,800.77 USD) for students in rental accommodations. The opportunity cost of the additional two years of study for a bachelor’s degree has been included. We used the minimum wage for a Bachelor’s Degree Graduate working as a Civil Servant in Thailand [12], which is THB15,000 (428.57 USD) per month, as a reference; as a result, the adjusted estimation is THB1,265,027 (36,143.63 USD) and THB1,823,027 (52,086.49 USD) for the student who lived at home and in a rental accommodation, respectively.

## Discussion

Surprisingly, for a Dental student’s education, tuition fees are the smallest portion of the overall expenses. The most significant expense is the living costs which can be considered extremely expensive for most students. The Faculty of Dentistry, Mahidol University, is located in central Bangkok and does not have a university dormitory for their students which profoundly affects the living costs for students having to stay in rental housing for the duration of their education. The average total yearly cost of living expenditure of a typical Thai Household is THB253,728 (7249 USD) [10]. In comparison, the estimated yearly spend for a dental student (living costs only) is estimated to be THB135,800–148,700 (3880–4249 USD) per year for a student who lives at home and THB198,482–260,300 (5,671–7,437 USD) per year for a student who resides in rental accommodations. Astonishingly, the yearly cost of living expenses of a dental student is at a minimum, is more than 54.2% of the typical total yearly expenditure of an average Thai household. This higher-than-normal expenditure can be financially devastating for the entire family. Therefore, we can reasonably assume that the average Thai family is not able to afford the high cost of a Dental Degree for their children, even in Public Universities. Notably, only 5.0% of students obtained additional financial support, and 65% of those students who received or sought financial aid came from families with household incomes above THB30,000 (1720 USD) per month. This data highlights the need to question and further evaluate the availability and equal access to current educational financial support schemes.

Arguably, the estimated cost in this study may in fact reflect the general cost of Higher Education in Thailand as a whole, since there is no comparison with a student expense in other fields of study. Although these findings have some uniqueness, such as the high cost of clinical uniforms and laboratory equipment (especially for 4th-year and 6th-year students who spent more on educational related expenses than others) and the increased costs related to the additional 2 years for a bachelor’s degree. Therefore, further study is needed to more accurately determine the differences between the cost of general Higher Education versus Dental education in Thailand.

The high cost attributed to medical and dental education is widely recognised internationally [1, 8, 13]. One of the most common negative effects due to the higher cost of Medical and Dental education is the accumulation of massive student debt, which has been an increasing socioeconomic issue in developed countries such as the U.S.A, U.K, Canada, and New Zealand for decades [1, 14–17]. Student debt can negatively affect dental students, dental schools, individual dentists, and the profession as a whole. For example, potential for enormous student debt could affect the students’ choice of dental school because some of them would not be able to afford the rent if they enrol in a school far from their current residence [1]. Student debt can also determine the career choices of dental graduates; for instance, they would be less likely to work in a rural area, primary care sector or as an academic [1, 15, 18, 19]. Moreover, it can potentially affect the clinical practices of dentists in debt by resorting to overbilling, or performing unnecessary treatment [8].

Another adverse effect due to the high cost of Medical and Dental education, is the skewing of socioeconomic diversity in the dental and medical workforce [7]. No studies have been conducted in Thailand assessing the root causes and effects of this phenomenon. In contrast, reports from other countries have suggested that reduced diversity in medical and dental schools can adversely affect the medical care of underserved populations. For example, recent studies from the U.S.A. have suggested that a more diverse background of medical students promotes a greater understanding of others from various cultural and economic backgrounds, thereby enhancing their ability to provide healthcare to people with various backgrounds. Physicians from a minority group are more likely to work in underserved communities [20, 21], which is a desirable outcome for any healthcare system.

Our study found that students from disadvantaged backgrounds are currently underrepresented. A low proportion of students come from a household income of less than THB30,000 (1270 USD) per month, only 6.4% of respondents fall in this category compared to an average 73.3% for Thai households in the same class (Table 5).

**Table 5:** Monthly household income of dental students and the national average. (*N* = 486).

Monthly Household Income (THB)	No. of Dental Students (%)	National (%)
<10,000 (<424 USD)	3 (0.61%)	20.9%
10,000–15,000 (424–635 USD)	3 (0.61%)	18.6%
15,001–30,000 (645.1–1270 USD)	25 (5.13%)	33.8%
30,001–50,000 (1,270.1–2118 USD)	73 (14.98%)	15.6%
50,001–100,000 (2,118.1–4235 USD)	214 (43.94%)	8.9%
>100,000 (>4235 USD)	168 (34.50%)	2.3%

*THB* Thai Baht, *USD* US Dollar, *No* number.

Inequities in the access to public dental schools can be attributed to the high cost of the Dental Degree, which affects the career choices of many high school graduates [13, 22] The enrolment method may also affect access, because they mainly rely on academic achievement [7, 23] in favour of students from an affluent background [24, 25].

One of the main issues of the dental workforce in Thailand is the maldistribution of dentists. Even though the dentist-to-population ratio was 1:12,000 against the target of 1:8,000, in some urban areas, the ratio was only 1:5000 compared to 1:16,000 in the rural areas [26]. Therefore, the high cost of dental education and the undiversified dental student could partly account for the dental workforce problem.

However, there were some limiting factors in this study. Notably, 78.5% of participating dental students came from two of the richest deciles of the Thai population. This might result in overestimating the actual cost of a Dental Degree in our study. Another limitation was the fact that we estimated the total cost of the six years in dental school by the yearly expenses of each student class. In a real-life situation, one student has to spend six years in dental school. Therefore, their living cost will be affected by the inflation rate. However, the data Ministry of Commerce indicates the average inflation rate in Thailand in the past ten years at around 1.3% [27]. Hence our estimations are similar to the actual figures.

### Recommendation

Even though the Thai government has provided access to very low-interest rate student loans for the entire duration of their academic studies, the maximum amount of loan a dental student can take out [5] is significantly lower than the current costs of the dental education. There is a need to increase support from governmental and non-governmental sources to make readily available more bursaries, scholarships, and interest-free loans for dental students. Needs-based financial assistance would greatly assist many prospective students, especially those from lower SES, to pursue a dental education and help them to reduce their financial burden.

Tuition fees of public dental schools are low compared to other countries [6, 28]. However, these fees can be a financial barrier for a student from a disadvantaged background wanting to pursue a dental career. The rising cost of tuition fees should be minimised to avoid the adverse effects mentioned above. It is particularly crucial because public universities transforming into autonomous universities in Thailand can inadvertently result in the increase of tuition fees, including afore mentioned dental faculties [29]. The fact that the Dental Faculty Consortium of Thailand agreed to raise tuition fees of all public Dental schools to THB100,000 (4193 USD) per year [30] will soon turn tuition fees into a significant barrier for students from disadvantaged backgrounds.

The diversity of students’ SES in dental school requires more attention even though the effects of inequitable SES on dental students in the Thai context needs further study for a better understanding of current and future impacts. However, from a social justice viewpoint, providing equitable access to students from underrepresented backgrounds is the obligation of public dental schools subsidised by taxpayers. A particular enrolment track should include SES background in the criteria, and the dental school enrolment policies should consider additional factors other than purely academic performance. We understand this issue can be politically challenging since providing what may be perceived as an advantage for poor students can reduce opportunities for other students, and accurately assessing a student’s socioeconomic background is a complicated task. Nevertheless, this issue is worth mentioning to spark further debate about the societal implications and cost of future dental and healthcare professional’s education.

## Conclusion

In this study, the estimated cost of an undergraduate Dental Degree is THB1,265,027 (36,143.63 USD) and THB1,823,027 (52,086.49 USD) for students living at home and renting accommodation, respectively. We have shown that the cost of Dental education in Thailand can be a significant financial burden, especially for students from disadvantaged (SES) backgrounds. Although these initial findings were from 2016, this was the first report which highlighted the issue prior to the Dental Faculty Consortium of Thailand agreeing to raise tuition fees of all public dental schools. In 2022, the tuition fees for a Thai undergraduate dental school in public universities was almost four times higher than tuition fees in the past decade. Therefore, the effects of the high cost of dental study in Thailand warrants further study, as well as identifying the cause and consequence of inequity and lack of dental student diversity pursuing higher education. This study was conducted to help forecast the financial impact of students in dental schools and provide a baseline for more in-depth analysis or research. We hope our study can facilitate much-needed attention to this issue and provide a footprint for further studies, providing equal dental education opportunities for all Thai students.

## Supplementary information

Appendix 1. Yearly living cost of dental student
