# Correction: Economic burden of becoming a dentist in Thailand

**DOI:** 10.1038/s41405-025-00359-z

**Published:** 2025-08-05

**Authors:** Teerawat Tussanapirom, Prachya Siribal, Phiranat Trirattanaphinthusorn, Witchapat Kengtong, Piyada Gaewkhiew

**Affiliations:** 1 Department of Community Dentistry, Faculty of Dentistry, Mahidol University, Bangkok, Thailand; 2 Chalermprakiat Hospital, Buriram, Thailand; 3 Private Dental Clinic, Bangkok, Thailand; 4 Department of Oral Medicine, Faculty of Dentistry, Chulalongkorn University, Bangkok, Thailand

**Keywords:** Dental foundation training, Dental clinical teaching

Correction to: *BDJ Open* 10.1038/s41405-023-00131-1, published online 10 February 2023

In this article the Ethics Approval Number ‘COA.NO.MU-DT/PY-IRB2016/004070’ should have been ‘COA. No. MU-DT/PY-IRB 2016/004.0701’. The original article has been corrected.

