# Supplementary material for: Economic burden of becoming a dentist in Thailand
Source: BDJ Open. 2023 Feb 10;9:5. doi: 10.1038/s41405-023-00131-1 (PMC9918459; doi:10.1038/s41405-023-00131-1)
Supplement: Supplementary file 1 — Appendix 1. Yearly living cost of dental student [file 41405_2023_131_MOESM1_ESM.docx]

Yearly living cost of dental student: Year 1

|  | food | transportation | social | etc | rent |
| --- | --- | --- | --- | --- | --- |
| N | 72 | 72 | 60 | 70 | 70 |
| Min | 27000.00 | 0.00 | 1200.00 | 5400.00 | 12000.00 |
| Mean | 83633.14286 | 1159.4429 | 8819.8 | 41605.1429 | 61419.42857 |
| Median | 75600 | 0.0000 | 3600 | 29094.0000 | 54000 |
| Std. Deviation | 31626.38999 | 2657.82235 | 10600.09 | 33774.89143 | 28769.14994 |
| Std. Error of Mean | 3780.076612 | 317.67053 | 1368.4657 | 4036.87165 | 3438.571107 |

Yearly living cost of dental student: Year 2-6 who live at home.

|  | food | transportation | social | etc | rent |
| --- | --- | --- | --- | --- | --- |
| N | 55 | 234 | 407 | 403 | 0 |
| min | 10800.00 | 0 | 1200 | 1200.00 |  |
| Mean | 36109.0909 | 12263.9872 | 30434.1007 | 46132.7643 | 0 |
| Median | 33600.0000 | 10800.0000 | 26400.0000 | 33000.0000 | 0 |
| Std. Error of Mean | 2651.15599 | 745.85792 | 962.47588 | 2019.40614 | 0 |
| Std. Deviation | 19661.49906 | 11409.43227 | 19417.22030 | 40539.29534 | 0 |

Yearly living cost of dental student: Year 2-6 who live at rental acomidation.

|  | food | transportation | social | etc | rent |
| --- | --- | --- | --- | --- | --- |
| N | 168 | 177 | 407 | 403 | 177 |
| Min | 14400.00 | 0 | 1200 | 1200 | 21000.00 |
| Mean | 72463.3631 | 6376.61 | 30434.1007 | 46132.7643 | 84120.68 |
| Median | 72000.0000 | 0 | 26400.0000 | 33000.0000 | 84000 |
| Std. Error of Mean | 1944.74915 | 796.6991 | 962.47588 | 2019.40614 | 2499.788 |
| Std. Deviation | 25206.82988 | 10599.39 | 19417.22030 | 40539.29534 | 33257.52 |

Yearly education-related expense of dental student. (N=486)

|  |  | Uniforms(฿) | Education Equipments(฿) | activities(฿) |
| --- | --- | --- | --- | --- |
| Year 1 | N | 74 | 72 | 470 |
|  | Min | 1100 | 150 | 400 |
|  | Max | 8365 | 4500 | 9200 |
|  | Mean | 3719.810811 | 1370.555556 | 2574.46808510638 |
|  | Median | 3182.5 | 1155 | 2300 |
|  | Std. Error of Mean | 198.5643928 | 108.6736959 | 69.0556522367603 |
|  | Std. Deviation | 1708.115493 | 922.1268876 | 1497.09086556122 |
|  |  |  |  |  |
| Year 2 | N | 105 | 101 | 470 |
|  | Min | 0 | 200 | 400 |
|  | Max | 3800 | 6300 | 9200 |
|  | Mean | 691.2380952 | 1532.782178 | 2574.46808510638 |
|  | Median | 500 | 1200 | 2300 |
|  | Std. Error of Mean | 60.5332599 | 102.9624363 | 69.0556522367603 |
|  | Std. Deviation | 620.2813339 | 1034.759678 | 1497.09086556122 |
|  |  |  |  |  |
| Year 3 | N | 71 | 70 | 470 |
|  | Min | 0 | 600 | 400 |
|  | Max | 6660 | 1150 | 9200 |
|  | Mean | 1860.140845 | 3501.857143 | 2574.46808510638 |
|  | Median | 1570 | 3325 | 2300 |
|  | Std. Error of Mean | 139.1223559 | 217.2505869 | 69.0556522367603 |
|  | Std. Deviation | 1172.265807 | 1817.648818 | 1497.09086556122 |
|  |  |  |  |  |
| Year 4 | N | 76 | 76 | 470 |
|  | Min | 1200 | 100 | 400 |
|  | Max | 16750 | 10500 | 9200 |
|  | Mean | 6596.578947 | 3940.789474 | 2574.46808510638 |
|  | Median | 6800 | 3800 | 2300 |
|  | Std. Error of Mean | 273.7905175 | 233.248814 | 69.0556522367603 |
|  | Std. Deviation | 2386.850395 | 2033.416018 | 1497.09086556122 |
|  |  |  |  |  |
| Year 5 | N | 49 | 48 | 470 |
|  | Min | 0 | 330 | 400 |
|  | Max | 5000 | 6900 | 9200 |
|  | Mean | 137.755102 | 2970.604167 | 2574.46808510638 |
|  | Median | 0 | 2850 | 2300 |
|  | Std. Error of Mean | 105.2464169 | 215.0281438 | 69.0556522367603 |
|  | Std. Deviation | 736.7249184 | 1489.75868 | 1497.09086556122 |
|  |  |  |  |  |
| Year 6 | N | 109 | 109 | 470 |
|  | Min | 0 | 560 | 400 |
|  | Max | 3000 | 35300 | 9200 |
|  | Mean | 192.2018349 | 6386.238532 | 2574.46808510638 |
|  | Median | 0 | 5000 | 2300 |
|  | Std. Error of Mean | 45.78836322 | 552.9892171 | 69.0556522367603 |
|  | Std. Deviation | 478.0445466 | 5773.376923 | 1497.09086556122 |
